# Biomarker-based early detection of epithelial ovarian cancer based on a five-protein signature in patient’s plasma – a prospective trial

**DOI:** 10.1186/s12885-021-08682-y

**Published:** 2021-09-16

**Authors:** A. Hasenburg, D. Eichkorn, F. Vosshagen, E. Obermayr, A. Geroldinger, R. Zeillinger, M. Bossart

**Affiliations:** 1 Department of Obstetrics and Gynecology, University Medical Center, Mainz, Germany; 2 Department of Obstetrics and Gynecology, Schwarzwald-Baar Clinics, Villingen-Schwenningen, Germany; 3 Department of Anesthesiology, Ortenau Clinics, Lahr-Ettenheim, Germany; 4 Department of Obstetrics and Gynecology, Medical University of Vienna, Vienna, Austria; 5 Section for Clinical Biometrics, Center for Medical Statistics, Informatics and Intelligent Systems, Medical University of Vienna, Vienna, Austria; 6 Department of Obstetrics and Gynecology, University Medical Center, Freiburg, Germany

**Keywords:** Ovarian cancer, Biomarker, Protein panel, CA125, Liquid biopsy

## Abstract

**Background:**

Trial on five plasma biomarkers (CA125, HE4, OPN, leptin, prolactin) and their possible role in differentiating benign from malignant ovarian tumors.

**Methods:**

In this unicentric prospective trial preoperative blood samples of 43 women with ovarian masses determined for ovarian surgery were analyzed. 25 patients had pathologically confirmed benign, 18 malignant ovarian tumors. Blood plasma was analyzed for CA125, HE4, OPN, leptin, prolactin and MIF by multiplex immunoassay analysis. Each single protein and a logistical regression model including all the listed proteins were tested as preoperative predictive marker for suspect ovarian masses.

**Results:**

Plasma CA125 was confirmed as a highly accurate tumor marker in ovarian cancer. HE4, OPN, leptin and prolactin plasma levels differed significantly between benign and malignant ovarian masses. With a logistical regression model a formula including CA125, HE4, OPN, leptin and prolactin was developed to predict malignant ovarian tumors. With a discriminatory AUC of 0.96 it showed to be a highly sensitive and specific diagnostic test for a malignant ovarian tumor.

**Conclusions:**

The calculated formula with the combination of CA125, HE4, OPN, leptin and prolactin plasma levels surpasses each single marker in its diagnostic value to discriminate between benign and malignant ovarian tumors. The formula, applied to our patient population was highly accurate but should be validated in a larger cohort.

**Trial registration:**

Clinical Trials.gov under NCT01763125, registered Jan. 8, 2013.

## Background

In 2016, the incidence of women newly diagnosed with ovarian cancer (OC) in Germany was 7350 with a morbidity rate of 17.6/ 100,000 per year (11.1 after standardization) [1]. Only 20% of patients are diagnosed at stage I and II because of missing screening strategies [2]. The combination of transvaginal ultrasound (TVUS) and laboratory test for cancer antigen 125 (CA125) in patient plasma for the early detection of ovarian cancer did not improve survival rates [3, 4]. Therefore German guidelines do not suggest screening for ovarian cancer (OC) [5, 6].

A good screening program for the early detection of ovarian cancer or its precursors and the presurgical differentiation between benign and malignant ovarian masses seen in TVUS is therefore sought-after. The International Ovarian Tumour Analysis Group (IOTA) published ten criteria which have shown high reliability in clinical diagnosis using TVUS for the differentiation between malignant and benign ovarian masses [7]. The IOTA prediction models showed excellent diagnostic performance with an Area under the Curve (AUC) of 0.96 and 0.95 [8].

CA125 is a validated therapeutic monitoring tool in ovarian cancer, but it is not sufficient to screen for ovarian cancer as it lacks specificity. It can also be increased in patients with endometriosis, pregnancy, other gynecological malignancies or inflammatory diseases of liver and pancreas [9]. Today it is the most important biomarker for epithelial OC in therapy monitoring [10]. Levels above standard value of 35 U/ml in blood samples are found in more than 90% of women with serous carcinoma of the ovaries in advanced stage disease.

Over the past decade different studies on biomarker panels as early diagnostic tools for OC were published. A common approach is the combination of CA 125 with other biomarkers aiming to improve its specificity concerning detection of OC [11–14].

Human epididymis 4 (HE4) is another biomarker overexpressed specifically in ovarian cancer. Unlike CA125 it is not elevated in endometriosis [15, 16].

Osteopontin (OPN) was initially detected as potential marker in osteoblasts. It is overexpressed in OC and in other tumors like lung-, breast- and colon cancer. Current research focuses on OPN as a diagnostic tool for OC [17, 18].

Leptin is a peptide hormone produced by fatty tissue, which is involved in the regulation of the sense of hunger and satiety. Leptin levels correlate directly with the amount of fat tissue in the body. Tissue hypoxia as seen in solid tumors can induce increase of leptin production. Various types of cancer show elevated leptin plasma levels, whereas reduced plasma leptin levels have been observed in ovarian cancer patients [19]. In breast cancer leptin is discussed as a novel diagnostic marker [20].

Prolactin is produced by the anterior pituitary gland and is responsible for differentiation of the mammary gland during pregnancy as well as for lactation. In OC patients prolactin might be elevated and plays a role in tumorigenesis [21].

Macrophage migration inhibitory factor (MIF) is a proinflammatory cytokine being involved in the complex process of regulating the innate and the adaptive immune system. It also affects tumorigenesis by inhibition of tumor suppressor p53 [22]. In 2007, Agarwal et al. showed increased plasma concentrations of MIF in OC patients [23].

Limited data exists regarding the combination of biomarkers to improve the early detection of OC. The ROMA (Risk of Ovarian Malignancy Algorithm) is a two-serum marker set of CA125 and HE4 to predict the malignancy of an ovarian mass [24]. .Gentry-Maharaj recently showed that adding HE4 to the OC screening with TVUS and CA125 does not improve specificity or sensitivity in the differential diagnosis of adnexal masses [25].

For the differentiation between benign and malignant ovarian masses we tested the presurgical plasma concentration of six biomarkers in patients with ovarian masses for their individual predictive value and for their potential within a diagnostic plasma protein panel.

## Methods

### Patient population

Patients with a suspicious ovarian mass were recruited from the Department of Gynecology at Freiburg University Hospital between July 2013 and July 2015. Patients have granted written informed consent prior to inclusion. The study was approved by the ethics commission of the University of Freiburg and is registered on Clinical Trials.gov under NCT01763125. Blood samples were taken within 24 h before surgery. Histological diagnosis of the surgically excised tumor ensured the correct allocation of the samples into the benign control and malignant group. Patients with tumor relapse or other cancer were excluded.

The isolation of cell and plasma fractions was achieved using density gradient centrifugation following the method set by Brandt and Griwatz (1996) [26]. The plasma samples were obtained after the two-layer density gradient centrifugation from the upper most layer. All samples were stored at − 80 °C prior to analysis.

### Multiplex analysis

Protein concentration of the plasma samples were determined using a multiplex analysis based on the Luminex Technology (Human Circulating Cancer Biomarker Magnetic Bead Panel 1; Millipore). The procedure is described by Pils et al. (2013) [14].

After thawing, the samples were diluted six-fold using the serum matrix provided in the kit. The quality controls and the lyophilized standard were reconstituted with 250 μl deionized water according to the manufacturer’ instructions. The working standards were prepared by diluting the reconstituted standard with assay buffer. After pre-wetting the assay plate with 200 μl assay buffer per well, each 25 μl 1:6 diluted samples, working standards, quality controls, and assay buffer as blank were added into duplicate wells. Then, each 25 μl of the mixed magnetic beads were added to each well and the plate was sealed and incubated with agitation at 4 °C overnight. Afterwards the plate was washed three times using 200 μl wash buffer using the magnetic plate washer. 25 μl detection antibodies were added to each well and incubated for 1 h at room temperature. In the next step, 25 μl Streptavidin-Phycoerythrin was added and incubated for another 30 min. The plate was then washed three times as described above. Finally 100 μl of sheath fluid was added to all wells. The plate was run in the Bio-Plex 200 array reader (Biorad). The Median Fluorescent Intensity (MFI) date were analyzed using a 5-parameter logistic curve-fitting method for calculating analyte concentration in the samples.

### Statistical analysis

Continuous variables are described by median and interquartile range (IQR). First, we examined the ability to discriminate between benign and malignant samples for all six proteins CA125, HE4, OPN, leptin, prolactin and MIF in univariate analysis using receiver operating characteristics (ROC) curves, summarized by the area under the curve (AUC). We computed 95% confidence intervals (CI) for the AUC based on 2000 bootstrap samples with the R package pROC. Second, we investigated the joint discriminatory ability by fitting a multivariable logistic regression model with elastic net penalty with the six proteins as explanatory variables and the malignancy as binary outcome variable [27]. This model was fitted with the R package glmnet, setting the elasticnet mixing parameter to 0.5 and choosing the tuning parameter by minimizing the leave-one-out cross-validated deviance [28]. For the multivariable analysis we took the log2 of the protein concentrations after adding 0.5. We evaluated the discriminatory ability of the multivariable model by calculating the leave-pair-out cross-validated c-statistic [29]. All statistical analyses were performed with R available at r-project.org.

Part of this data was published as a poster presentation at the Congress of the German Society of Obstetrics and Gynecology (DGGG) in 2020 [30].

## Results

### Patients’ characteristics

**Fig. 1:**
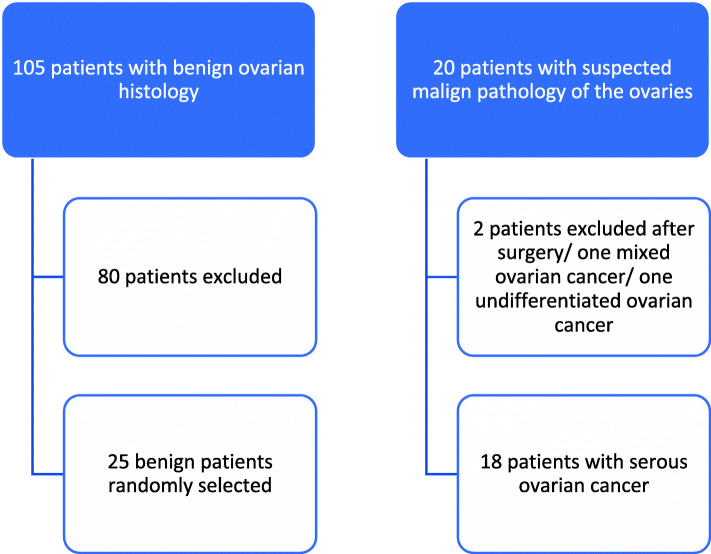
Patient recruitment. Patients were recruited from the Department of Gynecology at Freiburg University Hospital between July 2013 and July 2015. Data and samples of 43 of the patients were analyzed with an age range of 19 to 81 years

Of the 135 enrolled patients, 20 were diagnosed with ovarian cancer post-surgery, of whom 18 proved to have histologically serous ovarian cancer. Two patients were excluded after surgery, one having a mixed ovarian cancer and another having an undifferentiated ovarian cancer. Control group were 25 patients with benign histology, which had been randomly selected out of 105 non-cancer patients. Patient recruitment is displayed in Fig. 1. The median age of all 43 included patients was 53 years (range 19–81 years, IQR 17 years). All patient characteristics are displayed in Table 1.

**Table 1:** Patient Characteristics of the Freiburg Study Collective. Fédération Internationale de Gynécologie et d’Obstétrique (FIGO) staging based on Pecorelli et al. 1999 [31]

	Total	Benign	Malign
**Number**	43	25	18
Age Median ± IQR (Years)	53 ± 17	48 ± 20	59 ± 18
**Histology**			
Serous Cystadenoma	9	9	0
Endometriosis Cyst	5	5	0
Dermoid Cyst	6	6	0
Ovarian Fibroma	2	2	0
Other	3	3	0
epithelial ovarian cancer	18	0	18
**FIGO Stages**			
FIGO I-II	4	0	4
FIGO III-IV	14	0	14

### Single protein analysis

**Fig. 2:**
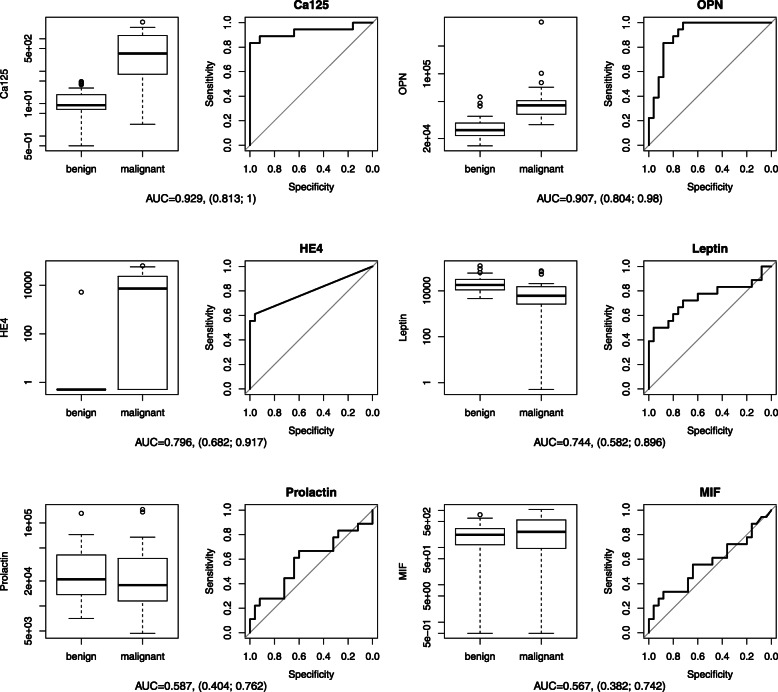
Boxplots comparing the expression in benign and malignant samples with corresponding receiver operating curve (ROC) for each protein. Two graphics are displayed for each protein. On the left side boxplots of benign versus malign samples are juxtaposed. The graphic on the right shows the corresponding ROC. Sensitivity and specificity differentiating benign from malign patients were applied at different threshold values. Below the graphics the area under the curve (AUC) for each protein is named with lower and upper limit for a confidence level of 95%

Blood samples were analyzed for CA125, OPN, HE4, leptin, prolactin and MIF. Boxplots and Receiver Operating Characteristics (ROC) Curves with corresponding AUC for each protein can be found in Fig. 2. AUC and median expression for each protein biomarker within the benign and malignant collective are displayed in Table 2.

**Table 2:** Single protein analysis for CA125, OPN, HE4, leptin, prolactin and MIF. Area under the curve (AUC) with 95% confidence interval (CI) and median expression plus interquartile range (IQR) of each protein biomarker in both the benign and malignant group are presented in the following table

Protein biomarker	AUC	with 95% CI	Median (IQR) expression / benign collective	Median (IQR) expression / malignant collective
**CA125**	0.929	(0.813–1.00)	9.02 U/ml (12.37)	348.96 U/ml (1053.90)
**OPN**	0.907	(0.804–0.98)	24,522.95 pg/ml (7934.32)	45,584.27 pg/ml (13,899.48)
**HE4**	0.796	(0.682–0.917)	0.00 pg/ml (0.00)	7349.02 pg/ml (22,947.18)
**Leptin**	0.744	(0.582–0.896)	17,867.52 pg/ml (20,264.12)	6025.32 pg/ml (11,297.58)
**Prolactin**	0.587	(0.404–0.762)	20,948.71 pg/ml (27,611.91)	17,912.65 pg/ml (23,332.14)
**MIF**	0.567	(0.382–0.742)	219.12 pg/ml (204.46)	262.59 pg/ml (429.09)

#### CA125

In the single protein analysis CA125 achieved an AUC of 0.929 (95% CI, 0.812–1.00). CA125 median expression of the samples with malignant histology of 348.96 U/ml was 38 times higher than in the benign collective with 9.02 U/ml.

#### OPN

For OPN an AUC of 0.907 (95% CI, 0.804–0.98) was calculated. With 45,584.27 pg/ml the samples with malignant pathology presented a significant higher median expression than the samples with benign histology showing a median expression of 24,522.95 pg/ml.

#### HE4

HE4 achieved an AUC of 0.796 (95% CI, 0.682–9.17). Median expression of HE4 in the malignant collective was 7349.02 pg/ml. In the benign collective HE4 concentration was below the detection limit for all but one person.

#### Leptin

Leptin had an AUC of 0.744 (95% CI, 0.582–0.896). Median expression of leptin at 6025.32 pg/ml was almost three times lower in samples with ovarian cancer compared to the control group with a median expression of 17,867.52 pg/ml. Our collective displayed a low sensitivity and specificity for leptin as a marker for OC.

#### Prolactin

AUC of prolactin was 0.587 (95% CI, 0.404–0.762). Median expression of prolactin in the benign group was slightly higher than in the malignant group with the factor 1.17. The mean value though was slightly lower with a factor of 1.02.

#### MIF

Median expression of MIF was similar in the benign (219.12 pg/ml) and the malignant tumors (262.59 pg/ml). MIF achieved an AUC of 0.567 (95% CI, 0.382–0.742).

### The 5-protein-panel formula to calculate the probability of a malignant ovarian tumor

Penalized logistic regression resulted in a multivariable model based on only 5 proteins (CA125, OPN, HE4, leptin and prolactin). According to the regression model the risk of any patient having a malignant tumor of the ovary can be calculated by inserting the plasma concentrations of the proteins in the formula below.

**Table:** 

Probability of a malignant ovarian tumor = (1 + exp (−(− 27.6311999 + log2 (Ca.125 + 0.5) * 0.6749108 + log2 (OPN + 0.5) * 1.9572380 + log2 (HE4 + 0.5) * 0.2234299 log2 (leptin+ 0.5) * -0.1320097 + log2 (prolactin+ 0.5) * -0.2910175)) ^ (− 1)	

**Fig. 3:**
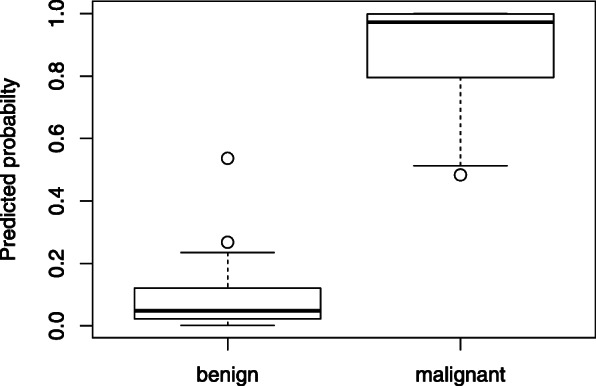
Boxplot display of predicted probability for a malignant ovarian tumor using the 5-protein-formula in the Freiburg collective

**Fig. 4:**
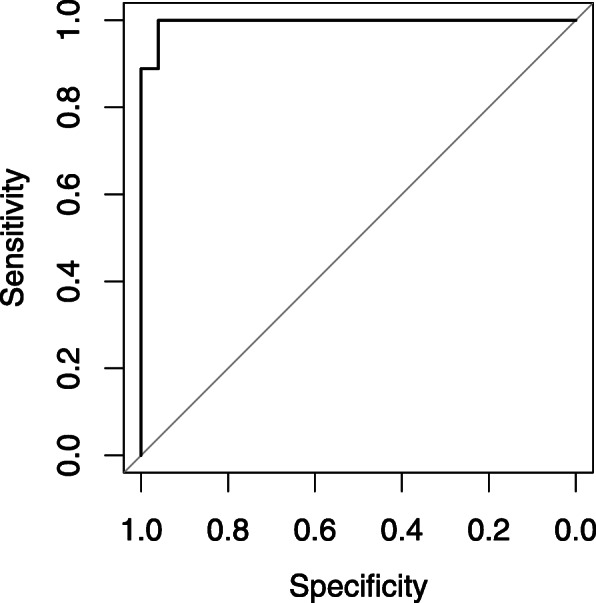
Receiver operating curve (ROC) of sensitivity and specificity using the model with the Freiburg collective. Area under the curve (AUC) = 0.996. After cross-validation the AUC was corrected to 0.96

The combination of plasma concentrations of the five biomarkers CA125, OPN, HE4, leptin and prolactin in the formula resulted in an AUC of 0.996. After cross-validation the AUC was corrected to 0.96. Boxplot and ROC are displayed in Figs. 3 and 4.

## Discussion

To date, there is no reliable diagnostic tool to differentiate between benign and malignant ovarian masses. Established tumor markers failed to provide screening benefits through early detection of ovarian cancer.

With this trial, we present a formula of five plasma markers to predict the malignancy of an ovarian mass as a highly accurate diagnostic tool. The protein panel of CA125, HE4, OPN, leptin and prolactin surpassed each single marker in its OC diagnostic capacity in patients with ovarian mass.

CA125 is a known highly predictive marker with an AUC of 0.929 for differentiating between benign and malignant ovarian tumors [29, 32–35].

Recent publications described OPN as a promising adjunct to CA125 in ovarian cancer screening tests [36]. Within our collective OPN with an AUC of 0.907 was highly accurate in differentiating between benign and malignant ovarian tumors. Our results here align with Moszynski et al. identifying an OPN/CA125 ratio as diagnostic tool in ovarian tumors [37].

HE4 has been reported to be superior to CA125 in differentiating ovarian masses, with low plasma levels in benign ovarian tumors. As CA125 plasma levels are already elevated in some patients with benign tumors of the ovaries, HE4 has a higher specificity for malignant ovarian tumors than CA125 [16, 38, 39]. Macedo et al. analyzed 45 publications on HE4 as diagnostic tool for OC. With an AUC of 0.916 HE4 was able to differentiate malignant or borderline tumors from benign tumors of the ovaries [40]. But HE4 plasma levels vary between histological tumor subtypes. Serous and endometrioid adenocarcinoma show elevated HE4 levels already in early stages of disease, while mucinous and clear cell subtypes in early stages only show a small increase of HE4 levels [24, 38]. Consecutive, HE4 is more qualified as a biomarker for serous and endometrioid carcinoma. Moore et al. compared HE4 and CA125 plasma levels of 1042 patients with benign and malignant tumors of the ovaries. HE4 levels were less elevated in patients with benign adnexal mass and outclassed CA125 [16].

Confirming previous research on leptin levels in OC, leptin was decreased in our OC group compared to the control group. With an AUC of 0.744, our investigations displayed leptin levels three times lower in the OC group than in the group with benign ovarian mass. Our results align with the decreased plasma levels of leptin in ovarian cancer patients [13].

Prolactin was not a valid single marker in OC diagnosis in our cohort with an AUC of 0.587. Prolactin levels already increased in benign adnexal tumors and therefore prolactin seems to be more selective in discriminating between the existence rather than the dignity of an adnexal tumor.

We developed a formula to estimate the presurgical likelihood of OC in a patient. With this formula a number between zero and one can be calculated using preoperative plasma levels of proteins in patient blood samples.

The combination of the above-mentioned five proteins (CA125, HE4, OPN, leptin and prolactin) in a protein panel surpassed the best single marker CA125 in its diagnostic capacity of OC. After cross-validation, the AUC of the panel amounted to 0.96 versus an AUC of 0.929 for CA125. It can be concluded that for our patient collective, the combined analysis of the biomarkers CA125, OPN, HE4, leptin and prolactin was more suitable for preoperative differentiation of ovarian masses than each single biomarker itself and can help the clinician to choose the optimal surgical treatment.

## Conclusion

To our knowledge this is the first identification of a predictive panel comprising of these five serum proteins. Several protein panels have been under investigation. Despite a high sensitivity in differentiating between patients with ovarian cancer and women without adnexal masses, many tests show a decline in discriminatory power when the control group has a benign ovarian mass [20, 23, 41]. A possible explanation could be the increase of plasma proteins secretion by benign ovarian tumors. In contrast, sensitivity of our protein panel, tested within a control group with benign ovarian tumors was able to reach an AUC comparably high as trials with a control group of women without an ovarian mass.

Our study has several limitations like the small sample size and the unicentric approach. Furthermore, we had no group without tumor or early stage ovarian cancer. Therefore, we cannot make a statement about the quality of our panel concerning detection of early OC. Due to the small sample size a correlation of the serum markers to FIGO stages cannot be made.

Taken together we identified a five-protein panel that can help clinicians to differentiate between benign and malignant ovarian masses in order to improve planning of surgical therapy and to improve counseling of patients before treatment. Using the protein panel in patients with ovarian tumors diagnosed via ultrasound could prevent unnecessary surgery for patients with benign ovarian mass. However, our panel requires further validation in a larger multicentric cohort with a control group of healthy women as well as women with early stage OC.

## Data Availability

The datasets used and/or analysed during the current study are available from the corresponding author on reasonable request.

## Abbreviations

AUC: Area under the Curve
CA125: Cancer antigen 125
CI: Confidence interval
DGGG: Deutsche Gesellschaft für Gynäkologie und Geburtshilfe
FIGO: Fédération Internationale de Gynécologie et d’Obstétrique
HE4: Human epididymis 4
IOTA: International Ovarian Tumour Analysis Group
IQR: Interquartile range
MFI: Median Fluorescent Intensity
MIF: Macrophage migration inhibitory factor
OC: Ovarian cancer
ROC: Receiver Operating Characteristics
ROMA: Risk of Ovarian Malignancy Algorithm
TVUS: Transvaginal ultrasound
